# Water Extraction Method Based on Multi-Texture Feature Fusion of Synthetic Aperture Radar Images

**DOI:** 10.3390/s21144945

**Published:** 2021-07-20

**Authors:** Wenbin Zhu, Zheng Dai, Hong Gu, Xiaochun Zhu

**Affiliations:** 1 School of Electronic Engineering and Optoelectronic Technology, Nanjing University of Science and Technology, Nanjing 210094, China; WenbinZhu@njust.edu.cn (W.Z.); guhong666@njust.edu.cn (H.G.); 2 School of Automation, Nanjing Institute of Technology, Nanjing 211167, China; XiaochunZhu@njit.edu.cn

**Keywords:** fractional dimension, synthetic aperture radar, texture feature, water extraction

## Abstract

Lakes play an important role in the water ecosystem on earth, and are vulnerable to climate change and human activities. Thus, the detection of water quality changes is of great significance for ecosystem assessment, disaster warning and water conservancy projects. In this paper, the dynamic changes of the Poyang Lake are monitored by Synthetic Aperture Radar (SAR). In order to extract water from SAR images to monitor water change, a water extraction algorithm composed of texture feature extraction, feature fusion and target segmentation was proposed. Firstly, the fractal dimension and lacunarity were calculated to construct the texture feature set of a water object. Then, an iterated function system (IFS) was constructed to fuse texture features into composite feature vectors. Finally, lake water was segmented by the multifractal spectrum method. Experimental results showed that the proposed algorithm accurately extracted water targets from SAR images of different regions and different imaging modes. Compared with common algorithms such as fuzzy C-means (FCM), the accuracy of the proposed algorithm is significantly improved, with an accuracy of over 98%. Moreover, the proposed algorithm can accurately segment complex coastlines with mountain shadow interference. In addition, the dynamic analysis of the changes of the water area of the Poyang Lake Basin was carried out with the local hydrological data. It showed that the extracted results of the algorithm in this paper are a good match with the hydrological data. This study provides an accurate monitoring method for lake water under complex backgrounds.

## 1. Introduction

Inland lakes are an important part of the biochemical and hydrological cycles of the earth, which are very vulnerable to climate change and human activities. The dynamic changes of water such as water scope, water level/depth, flow rate and water quality of the lake are closely related to flood or drought disasters, biodiversity and ecological protection, and are also closely related to human activities such as agricultural development and urbanization construction [1,2,3,4]. The traditional way to monitor lakes is to set up hydrological monitoring stations for observation. However, monitoring stations are few or inadequate in many remote regions, thus it is extremely difficult to carry out dynamic analysis and research on large-scale lakes due to limited observation information. Remote sensing is one of the most effective methods for monitoring lake water at present, since it has a fast response speed and wide field of view [5,6,7]. Since remote sensing images with long time series and largescales can be used to study the change of water areas dynamically, remote sensing technology is also of great significance in studying the rules of water areas [8,9].

Currently, water monitoring via remote sensing technology is mainly focused on optical satellite and synthetic aperture radar (SAR) satellite. Optical data such as Landsat and GF-2 can obtain multispectral images and NDVI indices, and have become commonly used in waterline mapping with its good spatial and temporal resolution [10,11,12,13,14]. However, due to the interference of cloud and light intensity, it is difficult for optical satellites to provide adequate time-series data to continuously monitor the change of water dynamically. In contrast, SAR such as a GF-3 remote sensor emits C-band microwaves to obtain wide range and high-resolution images throughout all times and all weather, and has a powerful imaging capacity of 12 modes to provide polarimetric images. Thus, SAR is more suitable for detecting dynamic changes in water monitoring [15].

The range of a lake is the most important hydrological element in monitoring the dynamic changes of lake water, which lays the foundation for flood warning, water resources planning projects, ecosystem services, wetland and biodiversity assessment and other important research directions [16,17,18]. Thus, the extraction of lake water is a key step in lake water monitoring. In recent years, many water extraction methods using SAR images have been proposed. The threshold method becomes a common method due to its high computational efficiency. Cazals et al. [19] used a threshold method to detect the hydrological information of coastal marshes based on S-1A data, and the accuracy approached 82%. Tian et al. [20] proposed an improved OTSU method to extract water area by comprehensively considering the variance of both internal and inner classes. Zhang et al. [21] proposed an adaptive threshold segmentation method based on wavelet energy and gradient. The wavelet energy features were used to perform rough segmentation of SAR images, and then the gradient threshold was used to perform precise segmentation of SAR images. However, the threshold-based method does not consider the spatial characteristics of SAR images, and it is difficult to select the optimal threshold in multiple time series SAR images, making the threshold method vulnerable to image noise and intensity inhomogeneity [22]. In addition to the threshold-based method, Shao et al. [23] reported that the K-means clustering method was used to identify barrier lakes from the background of SAR images. Leng et al. [24] used the fuzzy C-means (FCM) algorithm to obtain three classes of SAR images, namely water, background and middle region. Li et al. [25] used the active contour model (ACM) fitted by local Gaussian distribution to draw the dam and lake lines. Leng et al. [26] extracted the water body of Poyang Lake (PYL) in China using a local narrow-band ACM method. The second-order OTSU threshold method was first used for rough segmentation of SAR images. Then geometric active contour (GAC) method was used for accurate segmentation of SAR images, with a missed detection rate of about 0.71%. Although the reported methods and techniques are of great significance for the extraction and identification of lake water bodies, there exists some noticeable defects. For example, threshold-based methods and FCM algorithms generally have better effects only on simple water scenes. The GAC method is robust but requires a strict initial boundary, and normally has low computational efficiency for large-scale SAR images [27]. Therefore, it is necessary to develop a new method for water extraction from large-scale SAR images under complex backgrounds.

In this paper, Poyang Lake was selected as the main research area and SAR images were used to study the lake water dynamically. Poyang Lake is the largest freshwater lake in the Yangtze River Basin, with a complex environment such as folded waterline distribution in mountainous areas and dense buildings and human activities in urban areas. Thus, a water extraction algorithm was constructed to study the lake water and its change law by using the fused fractal features of SAR images. Since real images are fractal in nature [28], fractal models can characterize images with robustness. However, a single fractal dimension is normally not sufficient, thus many multifractal methods have been proposed in recent years for many image processing applications such as retina images [29], ultrasound images [30], multispectral images [31] and SAR images [32].

In our study, the fractal dimension and lacunarity were first calculated to construct the texture feature set. Then, the texture feature sets were fused into composite feature vectors using the Iterative Function System (IFS). Finally, the multifractal spectrum method was used to segment the water objects. Experimental results showed that the proposed algorithm accurately extracted the lake water of Poyang Lake. Compared with other water extracting algorithms, the proposed algorithm has a lower false alarm rate for Poyang Lake, Dongting Lake and Taihu Lake, and the least misjudgment caused by the speckle noise. The dynamic analysis of time-series SAR images in Poyang Lake combined with the local hydrological data shows that extraction results by the proposed algorithm are consistent with the local hydrological data, and the area of Poyang Lake changes sharply during the flood season.

## 2. Research Preparation

### 2.1. Research Area

**Figure 1:**
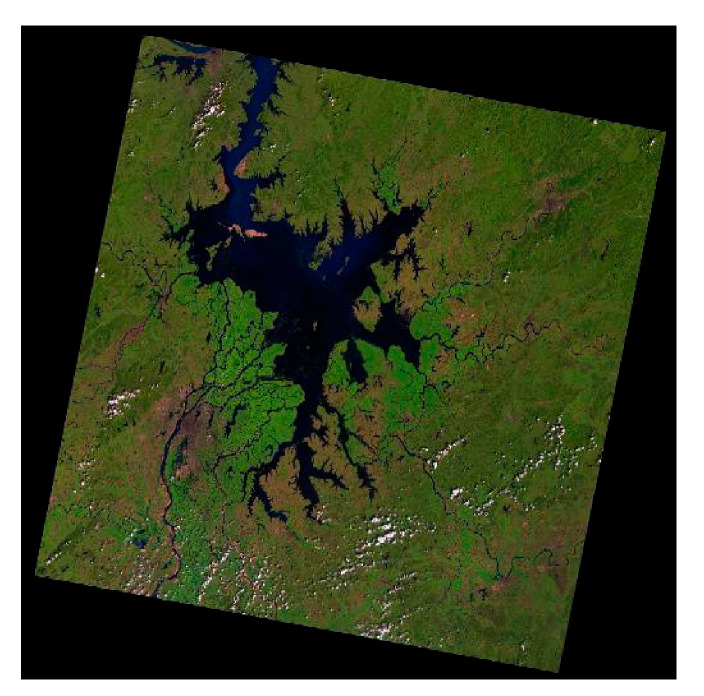
Remote sensing image of Poyang Lake.

The main research area is Poyang Lake in Jiangxi Province, China (Figure 1). Poyang Lake is the largest freshwater lake in China and a major hydrological subsystem in the middle of the Yangtze River Basin [33]. The water level of Poyang Lake varies greatly each year. During the rainy season (June to September), the area of Poyang Lake can reach 3500 km^2^. In the dry season (November to April), Poyang Lake covers less than 1000 km^2^, with only a few channels remaining. 

### 2.2. Dataset

All of the data studied in this paper are SAR images taken by GF-3 satellite. GF-3 SAR data is provided by the National Satellite Marine Application Service Center with the assistance from Dr. Peifang Wang’s team from Hohai university. The GF-3 satellite provides high spatial resolution, broad width, high accuracy, multiple modes SAR images with long working time. It is capable of acquiring C-band multi-polarization images with a resolution of 1–500 m and a width of 10 m–650 km. In our study, GF-3 satellites are observed in three modes: Fine StripMap I (FSI), Fine StripMap II (FSII) and Standard StripMap (SS). The resolution of SAR images is 5 m or 10 m. The degree of polarization of images is HH.

### 2.3. Data Preprocessing

In order to transform the original electromagnetic reflection data of SAR into images that are easily processed and can be compared quantitatively, the original data is preprocessed as follows: radiation calibration, complex data conversion, multi-view processing and image filtering. Firstly, the backscattering energy intensity of SAR images is converted into a backscattering coefficient, so that the images with different time phases are comparable. Next, the complex SAR data are converted into SAR amplitude data. Then, Envi V5.3 software is used for multilook processing and filtering of SAR data.

## 3. Water Extraction Algorithm

**Figure 2:**
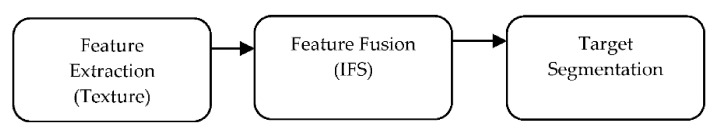
Flow chart of the proposed algorithm.

The primary problem of preprocessed SAR images in the process of water monitoring and dynamic analysis is the extraction of water. The water extraction algorithm proposed in this paper consists of three steps: texture feature extraction, feature fusion and target segmentation, as shown in Figure 2. The first step is to improve the extraction method of fractal dimension and lacunarity, then the texture feature set describing a water object is constructed. In the second step, the fractal dimension and lacunarity of an SAR image are fused into composite feature vectors by IFS. Finally, the multifractal spectrum method is used to realize target segmentation.

### 3.1. Extraction of Texture Features

**Figure 3:**
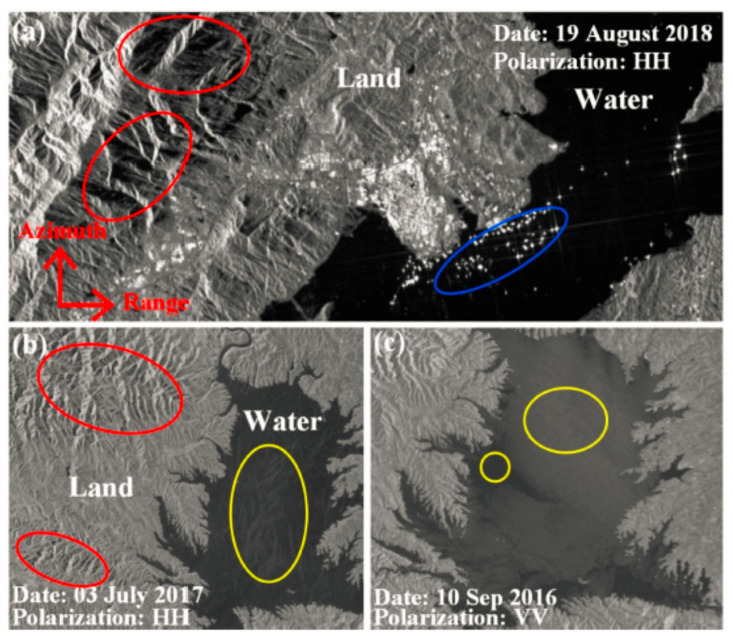
SAR image of Poyang Lake taken by GF-3 satellite. (**a**) SAR image of local regions of Poyang Lake obtained by HH polarization. The red circles are mountains, and the blue circle contains the boats on the water; (**b**) SAR images of local regions of Poyang Lake obtained by HH polarization. The red circles are the shadows and the yellow circle are the water surface; (**c**) SAR images of Poyang Lake obtained by VV polarization. The yellow circles show how water with different scattering characteristics could appear either bright or dark.

The background environment of the Poyang Lake region is complex, with many folded waterlines in mountainous areas, and dense human buildings and activities around the lake. Therefore, the water targets in SAR images are heavily interfered with. In general, quiet waters with a very low radar backscattering coefficient (CRB) are shown as black areas in SAR images, as shown in Figure 3a. Since the roughness of land is generally greater than that of water, water usually appears darker than land in SAR images. However, in some special cases, the backscattering intensity (IBR) of the water may be increased, which allows the water to appear brighter than land. In Figure 3b,c, the yellow circles indicate the water surface. The difference in brightness between the two images may be caused by wind, flowing water, or low wind speeds. In Figure 3a, the bright areas in the blue circle represent objects floating on the water. On the other hand, shadows in SAR images as shown in the mountain regions. In Figure 3a,b, red circles appear dark, and may lead to false extraction sincethe backscattering intensity is similar to that of water.

The fractal feature in texture describes the roughness of the object in the image, so it can better distinguish the artificial target from the natural target in the SAR image. The lacunarity feature represent the surface fluctuation characteristics, which are suitable for eliminating the interference of shadows in the mountainous environment. Therefore, in this paper the fractal dimension and lacunarity of SAR image texture features are selected to extract lake water.

Fractal features have inherent self-similarity, and random processes with self-similarity can be described by fractional Brownian motion. By establishing fractional Brownian random fields, the spatial distribution of random fields can be described. The preprocessed SAR image is defined as I(x,y). If the gray value of SAR image satisfies the discrete fractional Brownian random field (DFBR) field model, then the increment *r* to an arbitrary pixel $I(x_{0}-y_{0})$ is defined as:

(1) $$r=\sqrt{{\left(x-x_{0}\right)}^{2}+{\left(y-y_{0}\right)}^{2}},\Delta I(r)=\left|I(x,y)-I(x_{0}-y_{0})\right|$$

Since the increment of the DFBR field is a stationary process satisfying the average ergodicity, and the first and second order absolute moments of the increment of DFBR field are isotropic, then the *H* parameter can be expressed as:

(2) $$\begin{array}{l} H=H(r)\\ =\frac{\log(\frac{1}{N_{r}}\sum_{r>1}\left|I(x,y)-I(x_{0},y_{0})\right|)-\log(\frac{1}{N_{1}}\sum_{r=1}\left|I(x,y)-I(x_{0},y_{0})\right|)}{\log(r)} \end{array}$$

where $N_{r}$ is the number of pixels of distance *r* between I(x,y) and $I(x_{0}-y_{0})$. According to Equation (2), multiple data point pairs can be calculated, and the data point pairs can be fitted based on the least square method. The slope of the fitting line is the value of the H parameter.

According to the definition of fractal dimension, the fractal dimension of the SAR image in the DFBR model $D_{H}$ can be obtained by Equation (3).

(3) $$D_{H}=3-H$$

Since the self-similarity of SAR images can only be satisfied at a certain scale, we use the ε blanket method to extract fractal features when the self-similarity precondition cannot be satisfied, and use the measurement criteria to improve the calculation accuracy of the blanket method.

In the ε blanket method, the gray value of the pixels in the SAR image I(x,y) is regarded as the height information. Construct blankets of height 2ε areon both sides of the image plane I(x,y). The surface area of the predicted area is the blanket volume divided by 2ε.Therefore, for different ε blanket layers, the surface area can be calculated accordingly.

Assuming that the gray function of the SAR image is z=f(x,y), then the gray function z is a curved surface in three-dimensional space (x,y,z). If an appropriate scale is selected, two blankets covering the image surface from above and below can be constructed, and the area of the image surface can be determined by the volume of the upper and lower felt layers. The measured surface area varies with the selected scale. The coverage area of the double-layer blanket is:

(4) $$A_{\varepsilon }=\frac{\sum_{i,j}\left[u_{\varepsilon }(i,j)-b_{\varepsilon }(i,j)\right]}{2\varepsilon }$$

where $u_{\varepsilon }(i,j)$ and $b_{\varepsilon }(i,j)$ are the values of the corresponding positions of the upper and lower blanket, respectively. According to the measurement criterion, the exponential form of the fractal dimension *A*(*ε*) can be obtained:

(5) $$A(\varepsilon )=K\varepsilon^{d-D_{\varepsilon }}$$

where d is the topological dimension and K is the weighted constant. The fractal dimension $D_{\varepsilon }$ of the image is obtained by fitting logarithm and line:

(6) $$\log A(\varepsilon )=\log K+\left(d-D_{\varepsilon }\right)\log(\varepsilon )$$

In addition, we used the lacunarity feature to overcome the shadow interference in the lake waters (the red circle in Figure 3a). The lacunarity feature represents the fluctuation characteristics of the surface of the object and describes the intensity of texture change on the surface of the image, which is suitable for SAR images with a complex background.

For an SAR image I(x,y) of size N × N, assume that there is a sliding box of size L × L on the image window W × W. For each L × L sliding box, the maximum and minimum values of the pixels in the frame are Max(i,j) and Min(i,j) respectively, and the difference can be expressed as:

(7) δ(i,j)=Max(i,j)−Min(i,j)

Box quality $M_{i,j}$ is defined as the fluctuation degree of pixel intensity in the local area of the image:

(8) $$M_{i,j}=ceil\left[k\delta (i,j)/L\right]$$

where *k* is the weight coefficient. The probability function of box mass Q(M,L) is:

(9) $$Q(M,L)=\frac{n(M,L)}{{\left(W-L+1\right)}^{2}}$$

Then the lacunarity Lac on the scale *L* is:

(10) $$\mathrm{Lac}=\frac{\sum_{M}M^{2}Q(M,L)}{{\left[\sum_{M}MQ(M,L)\right]}^{2}}=\frac{\mathrm{var}(M)}{{\left[E(M)\right]}^{2}}+1$$

where *E*(*M*) and var(*M*) are the expectation and variance of *M*, respectively. From the above equation, the lacunarity of the center pixel of the image window can be obtained. By moving the W × W window across the entire image, the lacunarity for each pixel can be calculated.

### 3.2. Feature Fusion Based on IFS

In order to fuse the previously obtained fractal dimension $D_{H}$, $D_{\varepsilon }$, and lacunarity Lac into joint features for segmentation, an IFS-based method is proposed to construct feature vectors. Linear transformation of composite feature images is established to solve the problem that single feature can only meet the hypothesis conditions at a certain scale. Composite feature vectors representing images from multiple dimensions are constructed, which is conducive to the fusion of multiplefeatures for subsequent segmentation.

IFS is a finite set of compressed transformations on $R^{n}$: {w_i_, i = 1,…, N}. The transformation of a compact subset B ⊂ R^n^ is

(11) $$W\left(B\right)=\overset{N}{\underset{i=1}{\cup }}w_{i}\left(B\right)$$

The singularity A in W is an attractor of IFS, and can be obtained by any initial compact set B ⊂ R^n^:

(12) $$A=W\left(A\right)=\underset{k\to \infty }{\lim}W^{k}\left(B\right)$$

In this paper, we need to learn from the set of control points {(t_i_, x_i_), i = 0,1, …, N} to approximate the curve of the image boundary. These curves are usually fractal (continuous but nowhere differentiable), where theattractor is the graph of control points interpolated by the function f, i.e., for each i, f(t_i_) = x_i_. Although the structural solution cannot be obtained in general, Barnsley’s collage theorem gives an approximation that the attractor is close to the IFS of A.

For simple curves in R^2^, the affine IFS model is very convenient because all possible two-dimensional transformations can be described by affine transformations,

(13) $$w_{i}\left(\begin{array}{c} t\\ x \end{array}\right)=\left[\begin{array}{cc} a_{i} & 0\\ c_{i} & d_{i} \end{array}\right]\left[\begin{array}{c} t\\ x \end{array}\right]$$

When the following conditions are met:

$$\left\{\begin{array}{c} t_{0}<t_{1}<\ldots <t_{N}\\ a_{i}=\frac{t_{i}-t_{i-1}}{t_{N}-t_{0}}\\ \begin{array}{c} c_{i}=\frac{(x_{i}-x_{i-1})-d_{i}\left(x_{N}-x_{0}\right)}{t_{N}-t_{0}}\\ \left|d_{i}\right|<1 \end{array} \end{array}\right.$$

It can be proved that there exists a measurement where the mapping w_i_ is contracted and the attractor of IFS is a function graph of the interpolation points {(t_i_, x_i_), i = 0, 1,…, N}. The free parameter d_i_ is the scaling factor in the vertical direction and has the following relationship with the fractal dimension D of the curve:

(14) $$\overset{N}{\underset{i=1}{\sum }}a_{i}^{D-1}\left|d_{i}\right|=1$$

For the more complex boundary (high curvature boundary), we use the generalized fractal interpolation function as the attractor of IFS. Its data points are three-dimensional data {(t_i_, x_i_, y_i_), i = 0, 1, …, N}, and the IFS model is expressed as:

(15) $$w_{i}\left(\begin{array}{c} t\\ x\\ y \end{array}\right)=\left[\begin{array}{ccc} a_{i} & 0 & 0\\ c_{i} & d_{i} & 0\\ \begin{array}{cc} c_{\mathrm{yi}} & 0 \end{array} & 0 & d_{\mathrm{yi}} \end{array}\right]\left[\begin{array}{c} t\\ x\\ y \end{array}\right]$$

A form of c_yi_ can be obtained, by comparing the x_i_ in y_i_ instead of conditions; d_yi_ is the parameter that satisfies |d_yi_| < 1.

### 3.3. Target Segmentation

Since the feature vector set of the SAR image has been obtained, 2D-MFS and K-means are utilized to segment the target. Firstly, the two-dimensional singular power spectrum is extended by a Hölder Index. Then, the local MFS features are extracted and the image segmentation results are obtained by combining the local MFS features with a K-means method.

Let μ be a measure function on R^2^. For any point I(x,y) on the graph, let B(I,r) be the neighborhood of radius r around I(x,y). Then, the local density function $\alpha \left(I\right)$ at the point I(x,y), also known as the Hölder Index, is defined as:

(16) $$\alpha \left(I\right)=\lim_{r\to 0}\frac{\log\left(\mu \left(B\left(I,r\right)\right)\right)}{\mathrm{logr}}$$

In the previous section, the texture feature vector w of the SAR image is obtained, so replace μ with w in the above equation.

The power spectrum p corresponding to point I(x,y) can be obtained by following the following steps:

(1)  Define the minimum value $\alpha_{\min}$ and maximum value $\alpha_{\max}$ of the HölderIndex α(I), respectively $\alpha_{\max}=\max(\alpha (I))$, $\alpha_{\min}=\min(\alpha (I))$

(2)  Divide $\left[\alpha_{\min},\alpha_{\max}\right]$ into N parts, α_i_ = 1,2,…N represents the ith singular value, then the power spectrum p is:

(17) $$p\left(\alpha_{i}\right)=\underset{I\left(x,y\right)}{\sum }\alpha^{2}\left(x,y\right),\alpha \left(x,y\right)\in \left[[\alpha_{i},\alpha_{i+1}\right],i=1,2,\ldots ,N$$

Then the two-dimensional MFS (2D-MFS) of SAR image is obtained by following the ensuing method.

For any ϵ ∈ R, we define

(18) $$E_{\epsilon }=\left\{I\in R^{2}:\alpha \left(I\right)=\epsilon \right\}$$

where E_ϵ_ is the set of all the pixel points I(x,y) with local density ϵ. Then, 2D-MFS corresponding to point I(x,y) can be expressed as:

(19) $$f\left(\epsilon \right)=\left\{\lim_{r\to 0}\frac{\mathrm{logN}\left(r,E_{\epsilon }\right)}{-\mathrm{logr}},\epsilon \in R\right\}$$

where N(r,E_ϵ_) is the minimum value of radius r that can cover E_ϵ_.

**Figure 4:**
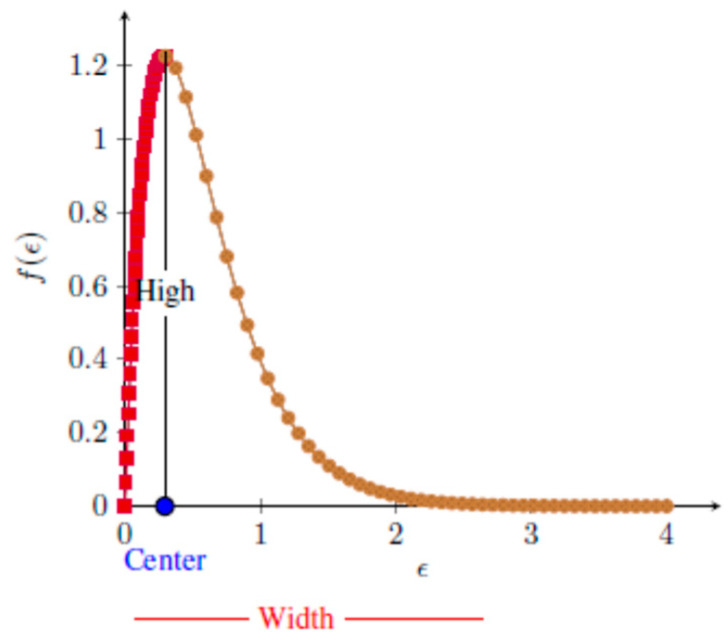
Diagram of the 2D-MFS curve.

Figure 4 is a reference diagram of the 2D-MFS curve. In Figure 4, four main properties of the 2D-MFS curve are shown: center ($\epsilon_{\mathrm{center}}$), maximum height ($f{\left(\epsilon \right)}_{\max}$), amplitude (width $f(\epsilon_{\max})-f\left(\epsilon_{\min}\right)$) and its symmetry ($\frac{\epsilon_{\max}-\epsilon_{\mathrm{center}}}{\epsilon_{\mathrm{center}}-\epsilon_{\min}}$). Since the global MFS method is complex to calculate, four scalar values (center, width, height and symmetry) of 2D-MFS are used here, and then a K-means method is used for clustering segmentation. The overall segmentation steps are summarized as follows:

1.  Calculate the 2D-MFS value f(ϵ) at point I(x,y) according to Equations (16)–(19).
2.  For each 2D-MFS, its four attributes (maximum, center, width and symmetry) are calculated; each attribute corresponds to an image, and then the four images are squared and added to generate enhanced texture images.
3.  The K-means method is used for clustering segmentation of the enhanced texture images.

## 4. Experiment and Discussion

### 4.1. Algorithm Verification

**Figure 5:**
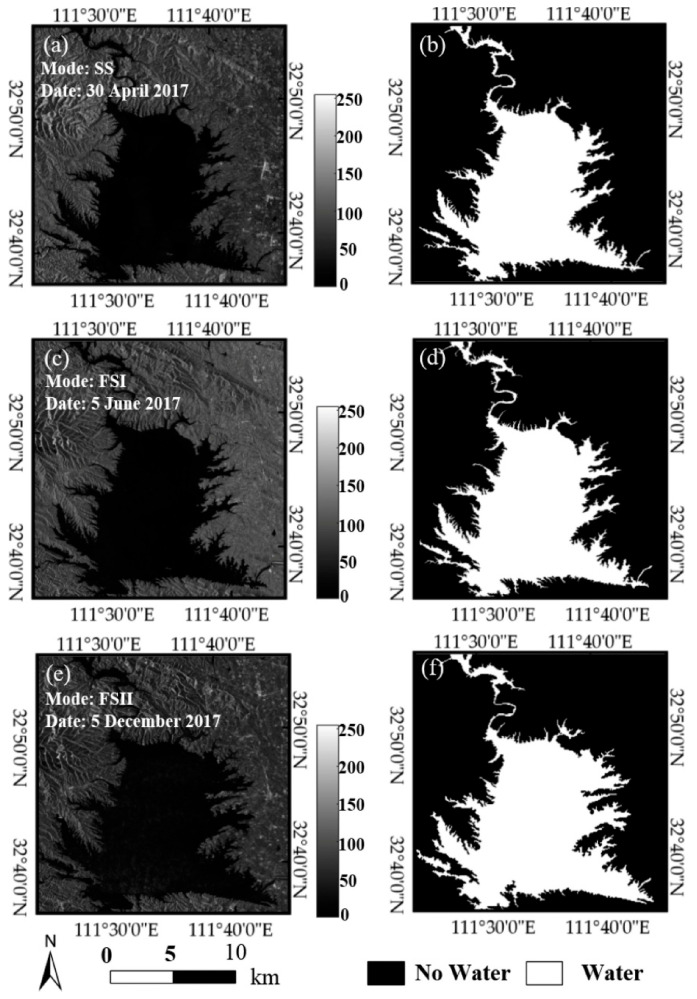
Water extraction results of GF-3 satellite SAR images in different imaging modes. (**a**,**c**) and (**e**) represent the original SAR images using SS (standard stripe), FSI (fine stripe 1) and FSII (fine stripe 2) imaging modes, respectively.(**b**,**d**) and (**f**) are the corresponding water extraction results obtained by using the algorithm in this paper.

To verify the algorithm proposed in this paper, three SAR images of Poyang Lake obtained by GF-3 satellite are tested. The GF-3 satellite can acquire C-band multipolar remote sensing images with a resolution of 1–500 m in 12 imaging modes. In order to verify the algorithm in this paper under different imaging modes, SAR images of the local area of Poyang Lake obtained under the imaging modes of SS (standard stripe), FSI (fine stripe 1) and FSII (fine stripe 2) were selected respectively, as shown in Figure 5a,c,e. Corresponding results obtained by using the algorithm in this paper are shown in Figure 5b,d,f, in which the white area is the water target obtained, while the black area is the background. As seen from Figure 5, the proposed algorithm can segment the SAR images of Poyang Lake correctly under different imaging modes, and accurately distinguish complex edges such as multi-forked tributaries.

### 4.2. Evaluation Metrics

In order to quantitatively evaluate and compare the accuracy and denoising capacity of the algorithms, this paper adopts the following three evaluation indexes:

(1) The F1 score is the weighted harmonic mean of precision, and ‘recall’ is used to measure the accuracy of the algorithms. ‘Precision’ is the fraction of the water pixels which are labeled correctly, and ‘recall’ is the fraction of all of the labeled water pixels that are correctly predicted. Thus, the F1 score is given as follows:

(20) $$F1=\frac{2\cdot \mathrm{precision}\cdot \mathrm{recall}}{\mathrm{precision}+\mathrm{recall}}$$

(2) False alarm rate (FAR) represents the ratio of dividing a non-water target into a water target. The closer the FAR value is to 0, the better the segmentation results become. A perfect image would give FAR = 0.

(21) $$\mathrm{FAR}=\frac{\mathrm{False}\;\mathrm{Postive}}{\mathrm{True}\;\mathrm{Postive}+\mathrm{False}\;\mathrm{Postive}}$$

(3) Equivalent Number of Looks (ENL) is a parameter of multilook SAR images, and multilooking is performed in order to mitigate speckle noise interference. Therefore, ENL is a measure of the noise intensity of speckle in an image, and its definition is as follows:

(22) $$\mathrm{ENL}=\frac{\mu^{2}}{\sigma^{2}}$$

where μ and σ are the mean value and standard deviation of an image, respectively.

### 4.3. Comparison and Analysis with Other Algorithms

**Figure 6:**
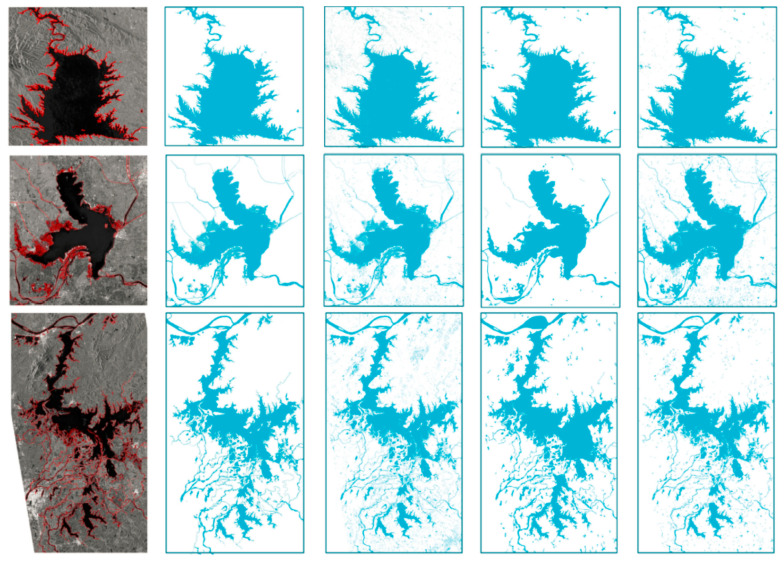
Comparison results of different water extraction algorithms. The first series of images are the original SAR images of Poyang Lake, Taihu Lake and Dongting Lake; the second column of images is the result of the algorithm in this paper; the image in the third column is the result of FCM; the image in the fourth column is the result of GAC; the image in column 5 is the result of MRF.

In order to validate the generality of the proposed algorithm, SAR images of Poyang lake, Taihu lake and Dongting lake were selected (image parameters are shown in Table 1), and the corresponding water targets were extracted by FCM [24], GAC [26], Markov random field (MRF) [34] and the proposed algorithm, respectively. MRF utilizes the contextual information of image pixels. In [34], it presents a PolSAR semantic segmentation method by employing 3D-DWT to extract multi-scale features and employing MRF to enforce label smoothness and alignment of label boundaries; thus, contextual information is fully used during segmentation. The F1 score, false alarm rate and equivalent appearance number were calculated and are shown in Table 2. The experimental results are shown in Figure 6.

**Figure 7:**
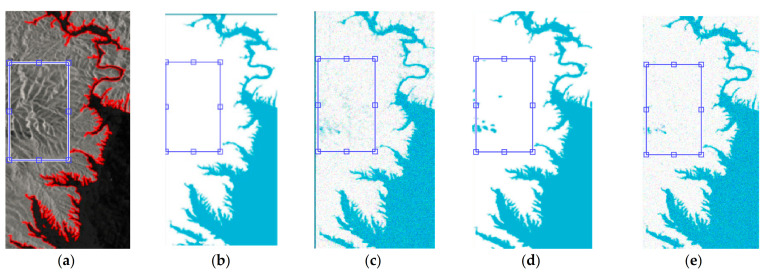
Enlarged view of comparison results of (**a**) the original image, (**b**) the proposed algorithm, (**c**) FCM, (**d**) GAC, and (**e**) MRF.

In Figure 6, the first column of images is the original SAR image, and the dark area is the water area. The second column of images is the result of using the algorithm in this paper. The third column is the results of images obtained using FCM; the image in the fourth column is the result of GAC, and its initial contour is from the result of FCM. The image in column 5 is the result of MRF. FCM, GAC and MRF methods fail to deal with shadows, and there is more mis-segmentation. Figure 7 is the local enlarged image of Figure 7a and the extracted results of each algorithm.

The area in the blue box in Figure 7 is the ridge area. It has been discussed that the shaded part of the mountain area is easily divided into the lake water body by mistake. In the segmentation results obtained by FCM, GAC and MRF methods, this area is wrongly divided into the water body, and only the proposed method can correctly judge this area. In addition, it can be seen from the amplified segmentation results that the noise in the image background is not completely removed by FCM, GAC and MRF methods, and the signs of noise can be clearly seen.

From the comparison of quantitative results in Table 2, it can be seen that the segmentation accuracy of the algorithm in this paper is the highest, and it is least affected by noise.

### 4.4. Dynamic Analysis

In order to study the dynamic changes of Poyang Lake, time series SAR images of the Poyang Lake region are analyzed. The proposed algorithm was used to extract and measure water bodies at different times in the local area of Poyang Lake in 2017. The overall contour of the lake extracted from different dates is consistent, but the waterline has obvious changes. In order to analyze the relationship between water line changes and upstream water inflow, the water body extraction results of SAR images were combined with the local hydrological data of the Poyang Lake region in 2017 to form statistical analyses of the water information of Poyang Lake. The results are shown in Table 3.

In Table 3, the lake area is analyzed by the water extraction results from SAR images, the lake perimeter is obtained from the local hydrological information, and the shoreline development coefficient is calculated by the area and perimeter. The shoreline development coefficient is defined as follows:

(23) $$D_{L}=\frac{L}{2\sqrt{\pi A}}$$

where L is the length of the shoreline, and A is the water area.

In order to compare the water dynamics of Poyang Lake during the rainy season, we compared the lake area and circumference from July to September 2017 with the lake area and circumference on 11 May 2017 (prior to the rainy season). According to the hydrological data, the Poyang Lake is in flood season in May, the Poyang Lake basin receives the maximum precipitation in June, and the Poyang Lake basin receives maximum flooding in late June and early July. On 30 July 2017 the water area of Poyang Lake reached the maximum, increasing by 5.8% compared with that of 11 May 2017.In August, the water situation of Poyang Lake was stable, so the water area was reduced. In September, the water area increased to the same level as that of 30 July 2017, indicating that there was a new upstream water potential in this region after August. Therefore, the water information extracted by the algorithm in this paper is consistent with the local hydrological data. Usually, when the water body area expands, the circumference also increases. However, the circumference of Poyang Lake decreases after the water body area increases, which may be due to the lake bank folding and filling phenomenon (effective within a certain range). The variation of the shoreline development coefficient also confirms this phenomenon.

## 5. Conclusions

In this paper, a water extraction algorithm from the SAR images of the lake water is proposed, which consists of texture feature extraction, feature fusion and target segmentation. Experimental results show that the proposed algorithm accurately extracts water targets from SAR images of different regions and different imaging modes. For the SAR images of Poyang Lake obtained by GF-3 satellite, the proposed algorithm is compared with FCM, GAC and MRF and shows significantly better accuracy than other algorithms, with an accuracy over 98% and a false alarm rate less than 3%. The proposed algorithm alsooutperforms other methods in the complex coastline area and mountain shadow interference. The time series analysis of water changes of Poyang Lake was carried out by SAR imagingalongsidethe local hydrological data in 2017. The results show that the water extracted by the proposed algorithm is consistent with the hydrological data. The water area changes around 6% and the perimeter up to 25%. This study demonstrates that the water area of Poyang Lake changes significantly during the flooding season, and the water area could change dramatically in a short period. The monitoring results lay a solid foundation for the preparation and prediction of flood control and drought prevention in this region.

## Figures and Tables

**Table 1:** SAR image parameters in Figure 6.

SAR Image	Area	Image Size (Pixel)	Polar	Date
A	Poyang	6152 × 6182	HH	6 May 2017
B	Dongting	5000 × 5373	VV	27 June 2017
C	Taihu	5000 × 8618	HH	17 July 2017

**Table 2:** Quantitative comparison of different water extraction algorithms.

SAR Image	Method	F1 Score	FAR (%)	ENL
A	Proposed	0.9923	0.31	4.28
	FCM	0.8847	6.86	2.82
	GAC	0.9010	1.97	2.75
	MRF	0.8655	4.59	2.52
B	Proposed	0.9912	1.01	4.55
	FCM	0.8825	13.87	3.37
	GAC	0.8513	16.32	2.50
	MRF	0.8463	15.09	2.26
C	Proposed	0.9854	2.87	8.05
	FCM	0.8068	19.97	5.38
	GAC	0.7865	19.61	4.06
	MRF	0.51	29.99	4.97

**Table 3:** Statistical results of water body change in Poyang Lake (compared with 11 May 2017).

Date	Perimeter/km	Perimeter Change Rate/%	Area/km^2^	Area Change Rate/%	Coastline Coefficient
11 May 2017	1219.4	0.0	1599.7	0.0	8.6
30 July 2017	911.7	−25.2	1692.5	5.8	6.3
1 August 2017	1068.3	−12.4	1588.4	−0.7	7.6
12 September 2017	1004.0	−17.7	1690.9	5.7	6.9

## Data Availability

Restrictions apply to the availability of these data. Data was obtained from the National Satellite Ocean Application Service Center and are available online (https://osdds.nsoas.org.cn/GaoFen (accessed on 1 May 2021)) with the permission of the National Satellite Ocean Application Service Center.
